# Development and Deployment of Air-Launched Drifters from Small UAS

**DOI:** 10.3390/s19092149

**Published:** 2019-05-09

**Authors:** Sara Swenson, Brian Argrow, Eric Frew, Steve Borenstein, Jason Keeler

**Affiliations:** 1 Ann and H.J Smead Aerospace Engineering Sciences, University of Colorado Boulder, Boulder, CO 80309, USA; brian.argrow@colorado.edu (B.A.); eric.frew@colorado.edu (E.F.); 2 Integrated Remote and In-Situ Sensing, University of Colorado Boulder, Boulder, CO 80309, USA; steve.borenstein@colorado.edu; 3 Department of Earth and Atmospheric Sciences, Central Michigan University, Mount Pleasant, MI 48859, USA; jason.keeler@cmich.edu

**Keywords:** unmanned research aircraft, UAS, airborne measurement technology, meteorology, atmospheric physics

## Abstract

Supercell thunderstorms can form extremely dangerous and destructive tornadoes. While high fidelity supercell simulations have increased the understanding of supercell mechanics to help determine how and when tornadoes form, there is a lack of targeted, in situ measurements taken aboveground in supercells to validate these simulations. Pseudo-Lagrangian drifters (PLDs) are atmospheric probes that can be used to attain thermodynamic measurements in areas that are difficult or dangerous to access, such as from within supercells. Of particular interest in understanding tornadogenesis is the rear-flank downdraft (RFD). However, strong outflow winds behind the rear-flank gust front (RFGF) make the RFD particularly difficult to access with balloon-borne sensors launched from the ground. A specific type of PLD, an air-launched drifter (ALD) that is released from unmanned aircraft systems (UAS), can be used to access RFD inflows, present at higher altitudes. Results from initial tests of ALDs are shown, along with results from a ground-released PLD test during a supercell intercept in the Oklahoma Panhandle on 12 June 2018. In characterization tests performed at the 2018 International Society for Atmospheric Research using Remotely piloted Aircraft (ISARRA) flight week, it was found that the ALD sensor system performs reasonably well against industry standards. However, improvements will be made to increase the aspiration of the sensor.

## 1. Introduction

Tornadoes are one of the most common and destructive forms of extreme weather in the United States. In 2017 alone, the United States experienced 1429 confirmed tornadoes [1], resulting in 35 tornado-related fatalities, 516 injuries, and over 649.18 million dollars in damages [2]. The deadliest and most destructive tornadoes occur in supercells. A region of descending air in the southwest quadrant of supercells known as the rear-flank downdraft (RFD) has been documented to play a critical role in the formation of tornadoes [3]. However, not all supercells produce tornadoes. It has been observed that tornadoes occur in 20 percent of supercells [4]. Therefore, a complete picture of the inner workings of supercells and the mechanics that lead to tornadogenesis is vital in accurately predicting when and where a supercell tornado will form. A better understanding of supercell tornadoes can help inform future research and increase tornado warning lead time.

Remote sensing systems such as weather radar do not provide thermodynamic data in the RFD. In situ measurements at the surface have been provided by mobile mesonets (e.g., Houston et al. [5]; Waugh [6]; Straka et al. [7]) and have shown promising results when used alongside radar data. A major benefit of the mobile mesonet (MM) is that, when an appropriate road network is available, the MM can move with the storm to target regions of interest, as the supercell evolves. However, even with increased mobility, the risk of placing an occupied MM beneath the most dangerous parts of the supercell can be unacceptable.

With the advent of unmanned aircraft systems (UAS), portions of the storm have been sampled that were otherwise inaccessible. UAS have allowed for temperature, pressure, and humidity measurements to be taken around and within vertical portions of supercells [5]. Facilitating the development of UAS has been a high priority in both the meteorological and engineering fields, in part driven by the advantages UAS pose in collecting aboveground in situ measurements from within supercells. Due to these technological advances, small UAS (sUAS) have been able to get closer to critical parts of the storm than ever before.

However, operating sUAS within supercell environments necessitates the ability to fly in dangerous conditions where rain, hail, and strong aerodynamic forces are present. These conditions risk the safety and operations of the aircraft, as well as the ability to conduct accurate measurements of the supercell. Therefore, direct measurements of the more severe portions of the supercell from sUAS are dangerous and ill-advised. It can be surmised that other methods for sampling above-ground portions of supercells are needed.

This paper is organized as follows. Section 2 describes the design of the pseudo-Lagrangian drifters, the sensor payload, and the method for the release of the pseudo-Lagrangian drifters from sUAS. Section 3 presents the simulations and field tests that have been performed on the pseudo-Lagrangian drifters. Finally, the discussion and conclusion are given in Section 4.

### Superpressure Balloons as Pseudo-Lagrangian Drifters

Pseudo-Lagrangian drifters have been used for decades to sample Earth’s atmosphere [8]. A “true” Lagrangian drifter has zero mass and perfectly tracks a fluid parcel as it moves through the atmosphere. In reality, it is impossible to create an ideal Lagrangian drifter, which has zero mass and an infinite drag coefficient. Therefore, the term pseudo-Lagrangian drifter is used to describe this form of atmospheric measurement.

Early atmospheric pseudo-Lagrangian drifters (PLDs) took the form of large, helium-filled, superpressure balloons that functioned as high-altitude observation platforms [8]. Unlike typical weather balloons, superpressure balloons are made of materials with minimal elasticity to maintain a relatively constant volume once fully inflated, enabling the balloon to maintain a fixed density altitude. This property makes superpressure balloons useful for measuring pressure variations and wind fields. Early PLDs were close to three meters in diameter and were used to measure oceanic wind currents [8]. The advent of miniaturized sensors has led to the reintroduction of PLDs for meteorological measurements [9] with a commensurate reduction in the size of the balloon required to reach a specific altitude.

During the Rivers of Vorticity in Supercells (RiVorS) project in May 2017, Markowski et al. [10] used ground-launched PLDs for thermodynamic measurements in supercells. Using ground-level inflows, the team was able to target the supercell forward flank region successfully. While the project had considerable success, the areas of the storm that can be targeted with this system is limited by the presence of ground-level inflows and outflows. Furthermore, variable-volume latex balloons compromise some of the Lagrangian aspects that can be leveraged to study the wind patterns within the supercell.

The rear-flank downdraft (RFD) is one of the regions of the supercell targeted for in situ measurements to study tornadogenesis [11]. The RFD is characterized by a downdraft that wraps around the mesocyclone. At the ground level, a strong gust front marks the boundary of the RFD outflow. This outflow pushes the air entrained by the RFD outwards from the southwest portion of the hook echo for an east-moving supercell.

**Figure 1:**
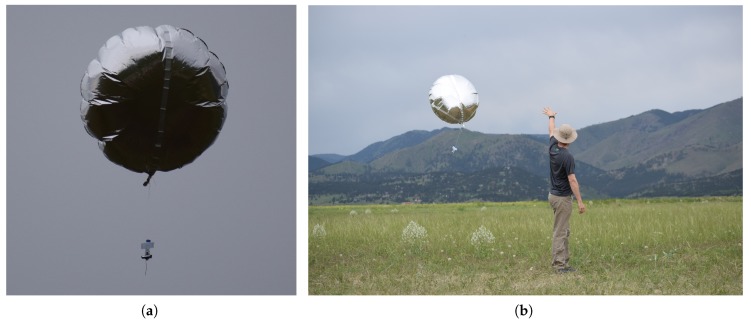
Pseudo-Lagrangian drifters (PLD). (**a**) PLD with attached microsonde. (**b**) Ground release of a PLD near Boulder, CO, USA. Photo credit: Roger Laurence, University of Colorado.

Outflows following the RFD gust front make ground-release of PLDs impractical as balloons will be carried away from the storm. Consequently, the RFD is a severely undersampled portion of supercell storms. However, supercell models indicate that air is entrained into the RFD at some altitudes above the ground. We expect PLDs to be entrained into the RFD if released at the appropriate altitude and distance from the storm. The motivation for the development of the air-launched drifter deployment system is to release PLDs to target supercell regions that cannot be accessed by ground release. Throughout this paper, the term PLD will be used to refer to this type of atmospheric measurement system in general, specifically those that are ground released, as in Figure 1. In contrast, air-launched drifters (ALDs) are a specific type of atmospheric PLD that are released from UAS.

## 2. Setup

### 2.1. Drifter Setup

The balloon envelope for the air-launched drifter (ALD) was comprised of 0.03 mm-thick polyethylene foil, a durable, lightweight material with properties that minimize the loss of helium lift gas through effusion; see Figure 1. The ALDs were designed to float at a maximum altitude of about 3 km above mean sea level (MSL). At and below this altitude, entrainment into the RFD was expected. By adding ballast to the ALD or decreasing the ALD balloon volume, the drifter altitude can be controlled before release. A 125-L balloon lifting a 92-g payload will reach a maximum drift altitude at 3 km MSL (using the 1976 standard atmosphere for density). Options for different volumes and shapes of balloons are currently being explored to maximize performance and minimize the amount of helium needed for each ALD. The sensor payload was connected to the bottom of the balloon with a 30-cm offset. The offset provided distance to mitigate signal interference with the balloon while still maintaining a semi-Lagrangian scheme.

**Figure 2:**
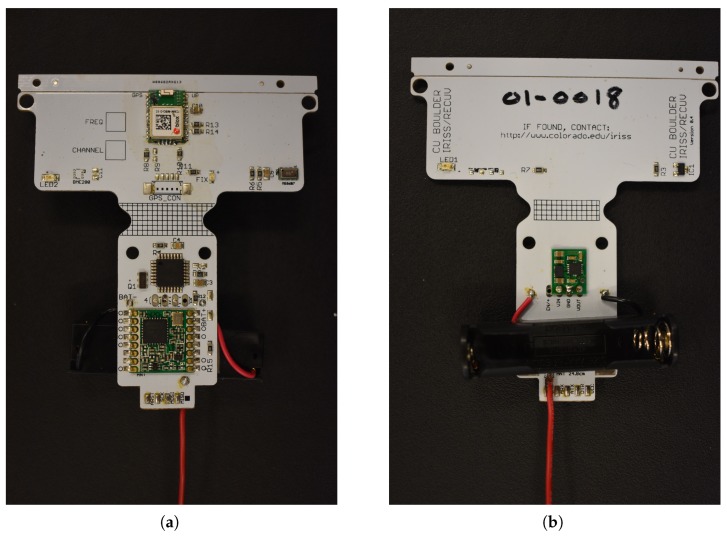
Closeup of the microsonde sensing system. (**a**) Front side of the microsonde; the GPS module, MCU, and radio module can be seen. (**b**) Back side of the microsonde; voltage converter and battery holder are shown. Onboard the microsonde are pressure, temperature, and relative humidity (PTH) sensors, a GPS, an MCU, and a transmitter. The sensor board is powered by a 1.5V AAA Lithium battery (not pictured). The microsonde is approximately 8 cm wide and 8.7 cm tall.

The sensor payload, displayed in Figure 2, was a microsonde developed at the University of Colorado’s Integrated Remote and In Situ Sensing Program (IRISS). The microsonde consisted of pressure, temperature, and relative humidity (PTH) sensors sampling at 1 Hz; a global positioning system (GPS) module to measure position, course, and speed; a microcontroller unit (MCU); and a 915-Hz, 100-mW transmitter. Table 1 and Table 2 summarize the component accuracy and range values. The weight breakdown of the entire ALD is described in Table 3.

The primary sensor onboard the microsonde, the MS8607 PTH sensor from TE Connectivity, was chosen as it combines high accuracy, low power consumption, and a compact design [12]. The first component of the sensor was a piezoresistive pressure sensing element, which measured both barometric pressure and temperature. The piezoresistive Micro-Electro-Mechanical Systems (MEMS) measured atmospheric pressure relative to a vacuum inside the MEMS, sealed by a thin membrane. A Wheatstone bridge was used to convert the pressure to a voltage output. This Wheatstone bridge also logged temperature by measuring the temperature-dependent resistance. The second component was a capacitive sensing element. A dielectric polymer film, sensitive to humidity, was placed between two electrodes. The absorption of moisture increased the sensor capacitance, and hence, a relative humidity measurement can be attained. Finally, a complementary metal-oxide-semiconductor application-specific integrated circuit (CMOS ASIC) was used for the digital conversion. The analog voltages of the pressure and temperature measurements were each output as a 24-bit digital value, and the relative humidity was output as a 12-bit digital value. These values were then sent to the Microchip ATmega328P MCU for processing [13]. The current draw of the PTH sensor ranged from a maximum of 1.25 mA during pressure and temperature signal conversion to a minimum of 0.03 μA during standby mode.

The CAM-M8Q GPS module from ublox is an omni-directional antenna module that can provide reception of up to 3 global navigation satellite systems (GNSS) (GPS, Galileo, GLONASS, BeiDou) at a given time [14]. The GPS module incorporated an integrated antenna design to be used in a vertical orientation. Furthermore, a ground plane integrated into the microsonde PCB improved signal reception. Operating in continuous mode, the module drew 28 mA at 3.0 V. This module gave latitude, longitude, altitude, and ground relative velocity measurements.

The microsonde was powered by a 1.5-V AAA battery. At room temperature, this battery was able to power the microsonde for approximately nine hours with decreased times expected at lower temperatures. A step-up synchronous DC/DC converter was used to adjust the battery output voltage of 1.5 to 3.0–3.6 V used to power the board.

While it is economically beneficial to recover and reuse the microsondes after deployment, it cannot be assumed that this will be possible. Therefore, onboard data storage is impractical and would take up a large portion of the limited mass budget available. Instead, the data were relayed to a ground station through the RFM95 HopeRF radio module [15]. Signal processing was controlled by the MCU. Data from the PTH sensor and GPS, as well as battery voltage were combined into a 20-byte binary packet that was transmitted to the ground station via the microsonde radio at a rate of 1 Hz. A cyclic redundancy check was performed on each packet by the microsonde radio such that only complete packets were output. The MCU used the GPS pulse-per second (PPS) output to time the transmissions during the allocated transmission slots. The current transmission period was limited to approximately 250 ms per packet. At a transmission rate of 1 Hz, the ability to synchronize transmissions was limited to four microsondes. The ground station used a 4-channel radio and a high-gain antenna to simultaneously communicate with up to four microsondes per channel; in total, communication with a maximum of 16 microsondes was possible. The current work is focused on optimizing the transmission process to maximize the number of microsondes with which a ground station can communicate.

### 2.2. ALD Deployment

The Mistral, a small unmanned aircraft system (UAS) developed by IRISS, outfitted for ALD release had the capability of performing a fill and release of three ALDs. Once all of the desired ALDs were released, the Mistral could travel to a location away from the storm to allow for safer control and conditions during landing. For in-flight deployment, it is necessary to carry the helium required to fill the ALD balloons onboard the UAS. A compact, high-pressure tank was used that had a volume of 1.5 L and had the ability to be pressurized to approximately 31 MPa. The helium provided by this tank was enough to fill three ALD balloons.

**Figure 3:**
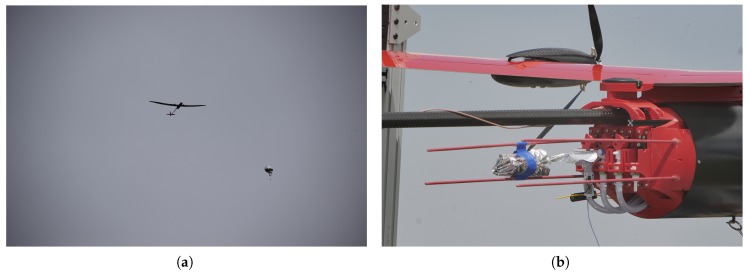
Images taken of the Mistral equipped to release air-launched drifters (ALDs). (**a**) ALD after release from the Mistral. Photo Credit: Roger Laurence, University of Colorado Boulder; (**b**) ALD in the storage position behind the fuselage of the Mistral before flight. Photo credit: Cole Kenny, Integrated Remote and In Situ Sensing Program (IRISS).

The helium tank was placed in the fuselage, forward of the center of gravity. Tubing connected the helium tank to the unfilled ALD balloons, which were located aft of the center of gravity. The ALD balloons were folded up along stabilizing rods that ran along the length of the aircraft towards the tail boom. Folds allowed the ALD balloons to fill up in a safe and controlled manner such that interference with control surfaces of the Mistral was minimized. The ALD balloons were connected to the helium tank through a quick disconnect connector. A servo rotated to press the connection between the tank and ALD balloons together such that the balloon fill process was initiated. Over a 60-s period, the ALD balloons were inflated behind the center of gravity of the aircraft. Positioning the ALDs aft of the center of gravity decreased the pitch induced on the aircraft as the balloon filled. Once the balloon reached the desired fill point, a release command was sent from the ground station. Once this happened, the servo rotated further to release the connector from its mount. The drag on the balloon was such that the ALD was pulled free and ascended away from the Mistral. This assembly is shown in Figure 3.

A FAA blanket certificate of authorization (COA) enabled the Mistral to fly up to an altitude of 400 ft. (120 m) AGL at class-G airspace anywhere in the United States. Other COAs that cover more than 500,000 sq. mi. (1.3 M km${}^{2}$) of the Great Plains have a 2500 ft. (760 m) AGL ceiling. The University of Colorado team is currently discussing a maneuver with the FAA that will enable the Mistral to briefly “pop-up” to 5000 ft. (1.5 km) to release the ALDs at a higher altitude above the supercell outflow and to increase the probability that the ALDs will ascend to the target equilibrium altitude before being entrained into the RFD. These release points are explored in the simulations of Section 3.1.

As of August 2018, a successful release of an ALD from the Mistral has been achieved. Current work is focused on reshaping the ALD balloon to minimize stress on the balloon during fill. It was observed during testing that the forces present behind the UAS were enough to occasionally tear the balloon membrane. Therefore, all sharp edges in the vicinity of the balloon were smoothed or removed. The stabilizing rods located near the balloons helped during the fill process, but it is believed that reshaping the balloon into a cylindrical design could aid in the success of fill and release of the ALD.

## 3. Results

### 3.1. ALD Trajectory Simulations

To assess the feasibility of deploying ALDs from an sUAS to be entrained into the RFD, simulations of possible ALD trajectories were performed. A high-resolution supercell simulation [16] was created using Cloud Model 1 (CM1), a three-dimensional, non-hydrostatic, non-linear, time-dependent, numerical atmospheric model developed at the National Center for Atmospheric Research (NCAR). The supercell simulation was integrated on a 240 × 192 × 20 km grid with 150-m horizontal grid spacing and 50-m vertical level spacing in the lowest 3-km AGL. Inclusion of radiative forcing, surface heat and moisture fluxes, and a semi-slip surface resulted in a storm environment consistent with realistic boundary layer turbulence.

**Figure 4:**
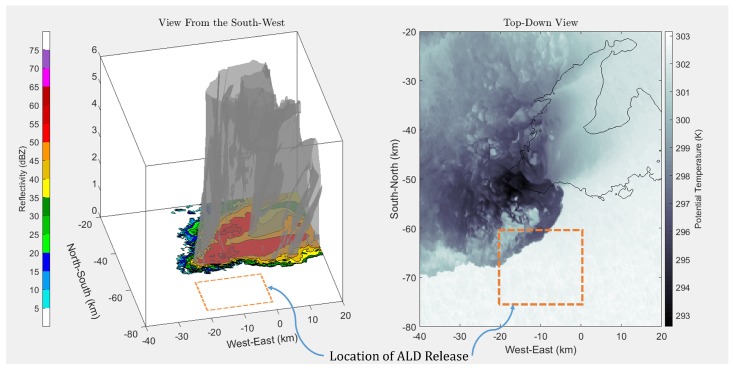
Simulation of drifters released into a north-east-moving supercell. On the left, a three-dimensional view of the ALD trajectory with respect to the 45-dBZ outline, a rough indicator of moderate rainfall within the supercell, is plotted above the supercell simulation reflectivity at the lowest level of the simulation. To the right are the same trajectories plotted at the altitude of release over the 45-dBZ outline in black, as well as the 2D slice of potential temperature at the altitude of release. Drifters were released approximately 2.5 km apart in both the east-west and north-south directions. The approximate location of ALD release is outlined in orange.

Simulated supercell data were provided in a fixed reference frame. At 16 GB per time frame, the supercell simulation data file was large enough to make repeated trajectory simulations computationally expensive. Therefore, to save on computation time, a single supercell frame was used to explore ALD trajectories. The mission concept of operations was to collect data from the drifters for a short period of time relative to the lifetime of the supercell, approximately one hour. ALD trajectories were simulated over a time frame of 120 min to help determine the importance of the sampling period. Future studies will employ time-dependent storm data to explore ALD trajectories. Figure 4 shows the simulated supercell without any calculated trajectories. The left plot gives the simulated reflectivity at the lowest level of the simulation, with the 45-dBZ surface contour plotted above. This 45-dBZ surface gave an approximation of where the edge of moderate rainfall associated with the storm is located. In the plot to the right, potential temperature is given along with the same 45-dBZ outline at the lowest level of the simulation. The potential temperature shown varied with the simulation altitude closest to the altitude of release to illustrate some of the prevalent structures.

The goal of the simulations was to determine not only if entrainment into the RFD was possible with ALDs, but where the ideal locations of release might be located. In this way, the sensing capability of each released ALD can be maximized.

The ALD trajectories were simulated by a time integration of Newton’s second law applied to the balloon system.

(1) $$m_{b}\frac{du}{dt}=\sum f_{ext}=\underset{\mathrm{weight}}{\underset{︸}{m_{b}g}}+\underset{\mathrm{buoyancy}}{\underset{︸}{m_{f}g}}+f_{\mathrm{added}\;\mathrm{mass}}+f_{\mathrm{drag}}$$

Here, u(t)=(u(t),v(t),w(t)) is the ALD velocity in the inertial frame, and:

(2) $$f_{\mathrm{added}\;\mathrm{mass}}=\frac{1}{2}m_{f}\frac{du}{dt}$$

is the force created, for a sphere, from the inertia of the air that is moved aside by the accelerating ALD. Furthermore,

(3) $$f_{\mathrm{drag}}=\frac{1}{2}\rho_{f}c_{D}A|u_{rel}{|}^{2}{\hat{e}}_{\mathrm{rel}}=\frac{1}{2}\rho_{f}c_{D}A\left|u_{rel}\right|u_{rel}$$

is the drag force from the relative velocity of air, where ***u***${}_{rel}$ is the relative velocity of the ALD with respect to the wind velocity. For the drag force, the area is the projected area:

(4) $$A=\frac{\pi d^{2}}{4}.$$

An (x,y,z) coordinate frame is employed, (x,y) describing the flat surface of the Earth and *z* in the vertical direction. From Equation (1), it follows that:

(5) $$\frac{du}{dt}=\frac{\left(m_{f}-m_{b}\right)g{\hat{e}}_{z}+\frac{1}{2}\rho_{f}c_{d}A\left|u_{rel}\right|u_{rel}}{m_{b}+\frac{1}{2}m_{f}}.$$

For simplicity, the body was approximated as a smooth sphere, and an approximation of the coefficient of drag $c_{d}$ from the Reynolds number based on the diameter of the balloon can be made [17]. For Red≤1:

(6) cd=24Red

1<Red≤400

(7) cd=24(Red)0.646

400≤Red≤3×105

(8) $$c_{d}=0.5$$

and 3×105<Red≤2×106

(9) cd=3.66×10−4(Red)0.4275

Equation (5) can be simplified by introducing:

(10) $$B=\frac{m_{f}-m_{b}}{m_{b}+\frac{1}{2}m_{f}}$$

and:

(11) $$K=\frac{\frac{1}{2}\rho_{f}c_{d}A}{m_{b}+\frac{1}{2}m_{f}}.$$

Then, the vector form of the equation of motion, Equation (5), can be rewritten as three scalar equations:

(12) $$\frac{du}{dt}=K\left|u_{rel}\right|\left(u_{w}-u\right)$$

(13) $$\frac{dv}{dt}=K\left|u_{rel}\right|\left(v_{w}-v\right)$$

(14) $$\frac{dw}{dt}=B+K\left|u_{rel}\right|\left(w_{w}-w\right)$$

or:

(15) $$\ddot{x}=K{\left[{\left(u_{w}-\dot{x}\right)}^{2}+{\left(v_{w}-\dot{y}\right)}^{2}+{\left(w_{w}-\dot{z}\right)}^{2}\right]}^{1/2}\left(u_{w}-\dot{x}\right)$$

(16) $$\ddot{y}=K{\left[{\left(u_{w}-\dot{x}\right)}^{2}+{\left(v_{w}-\dot{y}\right)}^{2}+{\left(w_{w}-\dot{z}\right)}^{2}\right]}^{1/2}\left(v_{w}-\dot{y}\right)$$

(17) $$\ddot{z}=B+K{\left[{\left(u_{w}-\dot{x}\right)}^{2}+{\left(v_{w}-\dot{y}\right)}^{2}+{\left(w_{w}-\dot{z}\right)}^{2}\right]}^{1/2}\left(w_{w}-\dot{z}\right).$$

Using a change of variables, Equations (15)–(17) can be rewritten as a system of first-order ordinary differential equations. Integrating with respect to time gives the projected trajectories of the ALDs. Calculations were carried out using the MATLAB ODE23 function. Wind velocity components were extracted from the supercell simulation and input as $u_{w}$, $v_{w}$, and $w_{w}$ in Equations (15)–(17). For the current iteration of the trajectory simulation, a search function was implemented to determine the supercell simulation point closest to that of the simulated trajectory. Wind velocity data from these points were dynamically found within the defined parameters of the ODE23 function call.

**Figure 5:**
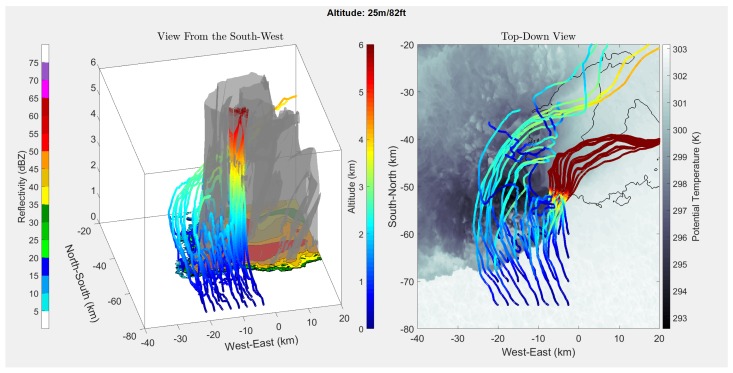
Simulated ALD releases 25 m above ground level, the lowest altitude of the supercell simulation. The colors of the trajectories correspond to the altitude of the drifters in both plots.

**Figure 6:**
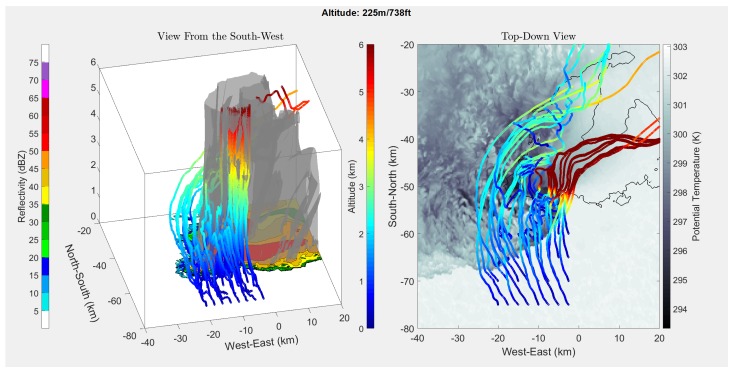
Simulated ALD releases 225 m above ground level.

**Figure 7:**
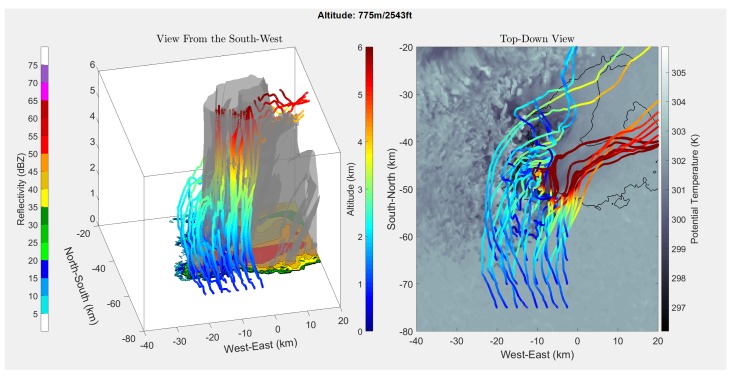
Simulated ALD releases 775 m above ground level, the altitude closest to the maximum ceiling of the certificate of authorization (COA) over the Great Plains.

Figure 4, Figure 5, Figure 6 and Figure 7 show the results of running the simulation at different release altitudes. The plots give the trajectories of a theoretical drifter released at 2.5-km horizontal intervals to the south of the storm, as can be seen by the orange outlines in Figure 4.

From the results, it appeared that the ALDs were most likely to be entrained when they were released within 5 km of the rear-flank gust front. This can be seen in Figure 5, Figure 6 and Figure 7 by following the ALD trajectories. In the trajectories that produced favorable entrainment, the ALD trajectories can be seen to appear as lighter blue as the ALD passed over the gust front. Following this, the trajectory returned to the original dark blue, indicating that the ALD was descending and had become entrained into the downdraft. Finally, many of these favorable trajectories indicated that the ALD traveled horizontally away from the RFD, entrained in the outflow. It is believed that these favorable trajectories will produce the most helpful data for studying the RFD. When the ALD was released too far south or west of the RFD, the ALD was pushed up and over the RFD into the updraft. These results support the expectation that there are release points upwind of the gust front where the ALD will rise above the horizontal outflow boundary where it will become entrained into the RFD. It is worth noting that other approximations for the drag coefficient [18,19] also produced results that suggest entrainment into the RFD.

In Figure 5, Figure 6 and Figure 7, it appears that at a release altitude of 25 m, 8 release points resulted in entrainment in the downdraft; at 225 m, 10 release points resulted in entrainment; and at 775 m, 7 release points resulted in entrainment. Therefore, the results show that it is possible to deploy ALDs for in situ measurements in the descending portion of the RFD. It is difficult to determine the relative added benefit of UAS release from these simulations as wind data were not available under 25 m. It was observed that there was some sensitivity to where the ALDs were released. At lower altitudes, if they were released too far to the southwest of the gust front, they were more likely to be pushed out around the outer portion of the storm. However, at higher altitudes, it becomes more likely that they will be pulled into the updraft and away from the RFD. It is expected that a combination of knowledge gained from these simulations, as well as field experience will enable release strategies for reliable penetration of the ALDs into the RFD.

Simulation results provided additional groundwork for performing similar analysis in the field. To assist targeting locations of ALD release, it should be possible to run these same simulations using radar data from nearby stations. Doppler radars use pulse pair motion to calculate precipitation motion. From this, an estimate of the wind field can be estimated. For use in the field, this could be used as an input to the simulations. Results could determine whether or not a given supercell is feasible for ALD release to target the RFD or other regions of the supercell. Furthermore, once feasibility is determined, results could aide pilots and scientists in establishing the ideal storm-relative locations of ALD release.

### 3.2. Initial Field Results

**Figure 8:**
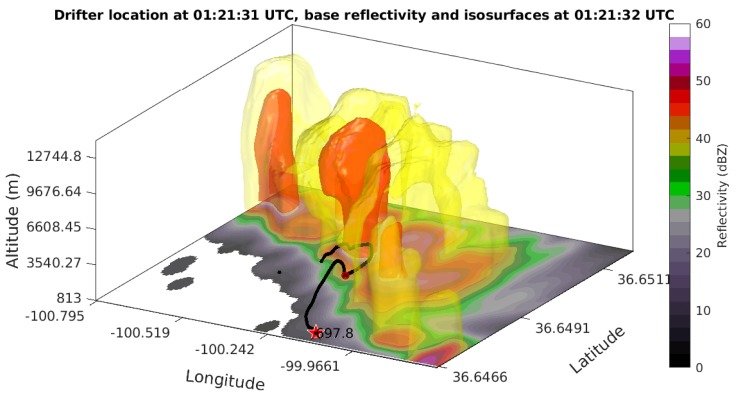
PLD data from the ground release done on 13 June 2018 in the Oklahoma Panhandle. The PLD was ground-released to the south of a convective storm and proceeded to move to the north-west. The PLD path, shown in black, overlaid on Next-Generation Radar (NEXRAD) data, transects the path of the supercell. The red star indicates the position of the PLD at time of release, with its altitude labeled in meters. The dark red dot indicates the point where communication was lost with the PLD.

**Figure 9:**
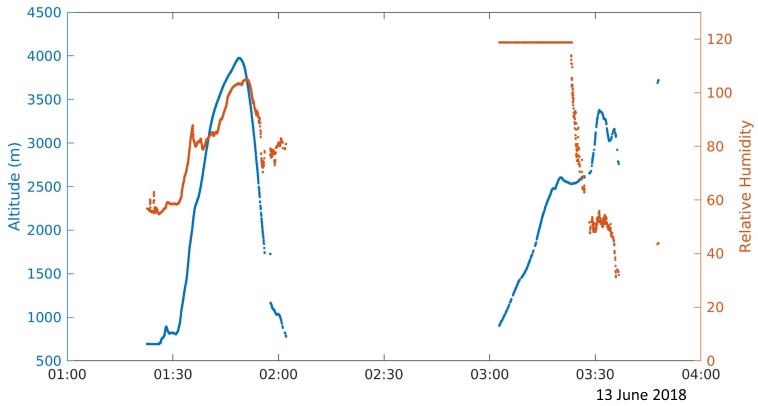
Altitude (blue) and relative humidity data (orange) from the PLD ground release into the 13 June supercell suggest the PLD could have been forced down due to the presence of precipitation in the supercell.

During the 2018 deployment of the Severe-storm Targeted Observation and Robotic Monitoring (STORM) project, a PLD was ground released into a supercell near Gate, Oklahoma. The PLD path through the supercell can be seen in Figure 8. It appeared that the PLD ascended to an altitude of 3.5 km MSL about 30 min after release before dropping back down to the ground where contact with the PLD was temporarily lost. The PLD was likely forced down due to the presence of a downdraft or due to the weight of precipitation. In Figure 9, it can be seen that when communications were reestablished, the relative humidity sensor was saturated. However, after the supercell passed, it is believed that a combination of a decrease in precipitation and increase in temperature caused the PLD to ascend to higher altitudes. It was seen that as the PLD ascended, it turned back towards the direction of storm movement southwards. During the ground release, communications were tested up to a range of 100 km, and the microsonde was able to transmit measurements for a period of two hours.

### 3.3. Microsonde Characterization Tests

Characterization tests of the microsondes were carried out during the Lower Atmospheric Process Studies at Elevation—a Remotely-Piloted Aircraft Team Experiment (LAPSE-RATE), in Alamosa, Colorado, during the week of 14 July 2018.

**Figure 10:**
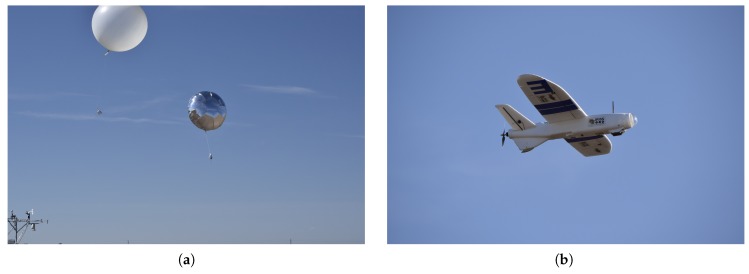
Microsonde characteristic test setups. (**a**) The microsonde was tested onboard a ground-released pseudo-Lagrangian drifter (PLD) (right). Results were compared to a Vaisala RS92-SGP onboard a National Severe Storms Laboratory (NSSL) weather balloon (left). Photo credit: Roger Laurence, University of Colorado; (**b**) Further tests were done onboard the Talon sUAS. Results were compared to an onboard Vaisala RS92. Photo credit: Roger Laurence, University of Colorado.

**Figure 11:**
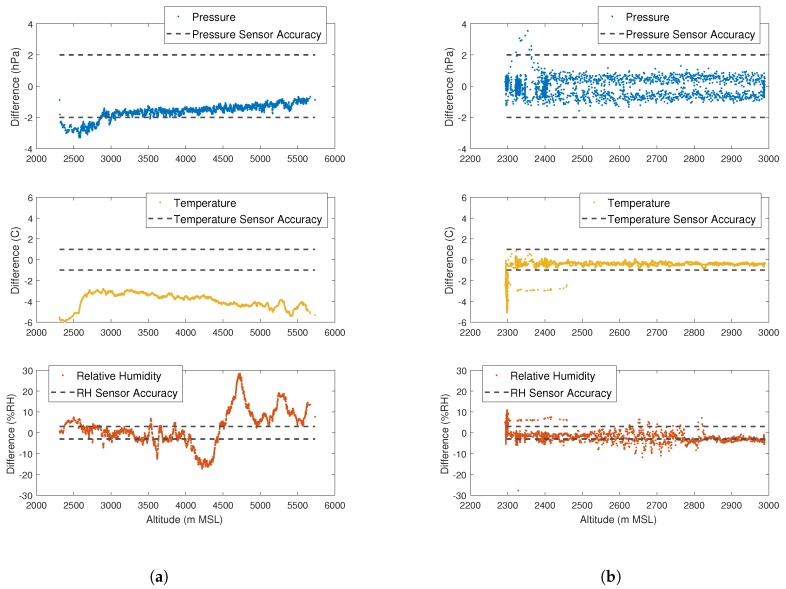
Vaisala RS92 and microsonde altitude comparisons in meters above mean sea level (MSL). (**a**) Comparison between the Vaisala RS92-SGP radiosonde on the NSSL weather balloon and the microsonde on a ground-released PLD. Vaisala RS92-SGP radiosonde data courtesy of Dr. Sean Waugh, NSSL; (**b**) Comparison between the microsonde flown alongside a Vaisala RS92 onboard the Talon sUAS.Validation trajectory

**Figure 12:**
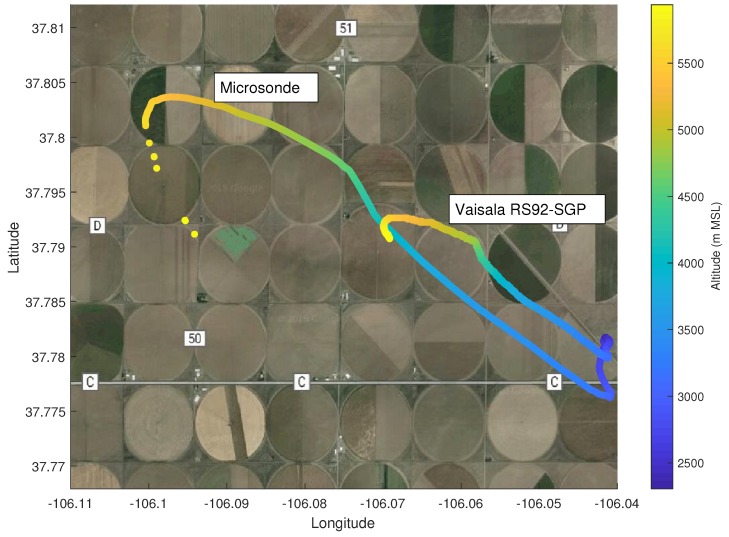
Trajectories of the Vaisala RS92 radiosonde on the NSSL weather balloon and the microsonde on a ground-released PLD.

For the first test, PLDs were ground released next to a Vaisala RS92-SGP attached to a National Severe Storms Laboratory (NSSL) weather balloon, as shown in Figure 10a. Results from the comparison between the microsonde and RS92-SGP are given in Figure 11a. It can be seen that for all three measurements, readings between the microsonde and RS92-SGP differed at magnitudes much larger than the expected sensor accuracy. This result was expected as the difference can, in part, be attributed to the manner in which the two systems profile the atmosphere. As the NSSL weather balloons acts as a typical radiosonde, it ascends at a relatively fast and somewhat constant rate. On the other hand, the PLD ascends at a slower rate and levels off as it reaches the target altitude. Immediately following release, the weather balloon and PLD followed similar trajectories. However, after less than a minute, the weather balloon and PLD were measuring very different parcels of air, as can be seen by comparing the two trajectories, as shown in Figure 12. As a result, the differences between the two sensors fell outside the quoted accuracy bounds (reported in Table 1) at higher altitudes. Since atmospheric temperature and pressure are functions of altitude and affected less by the specific parcel of air that is measured, the pressure and temperature differences appeared to be less sensitive to the path differences between the weather balloon and PLD. However, as can be seen in Figure 11a, there did appear to be a bias between the two measurement systems. Tests were done at high altitudes during a sunny week in July. Therefore, it is believed that radiative heating of the microsonde could have led to sensor bias. It is unlikely that radiative heating will have noticeable impacts on sensor measurements in supercell environments; however, a new version of microsondes is under development to shield the sensor from fluxes in board temperature due to radiative heating.

To get a better idea of the microsonde performance, a microsonde was flown next to a Vaisala RS92 under the wing of a Talon fixed-wing, foam-body sUAS. Results from the comparison between the microsonde and RS92 are given in Figure 11b. In this case, the differences between sensors appear to be much smaller throughout the flight. A spike can be seen in the pressure and relative humidity comparison data at the lowest flight altitude, around 2300 m MSL. This spike can be attributed to times prior to take off of the Talon and following landing. Achieving adequate airflow over temperature and relative humidity measurements is an important aspect in collecting accurate results [20]. It is expected that proper aspiration of both the microsonde and Vaisala RS92 was not attained before takeoff and after landing, when the spike in the comparison occurred. Therefore, work is underway to increase airflow over the sensors to achieve proper aspiration even when operating as a perfect Lagrangian drifter.

## 4. Conclusions

The air-launched drifter (ALD) system was designed to place pseudo-Lagrangian drifters (PLDs) into the rear-flank downdraft (RFD) of tornadic supercells to perform thermodynamic measurements of the RFD. PLDs offer a unique solution to sample the RFD safely and remotely. While ground-released pseudo-Lagrangian drifters have had promising success in measuring portions of the forward-flank downdraft, it is believed that the airborne Lagrangian drifter system would offer the freedom to sample more parts of the storm, including the RFD.

Simulations were done using high-resolution simulated supercell data to determine the feasibility and o guide the concept of operations for an ALD system. It was found that by releasing the ALD south of the storm, that they were most likely to become entrained into the RFD. These simulations are important in the understanding of supercell structures; however, it will also take field experience to understand fully how to successfully entrain the ALDs in the RFD. Furthermore, these simulations assumed that the supercell was in a steady-state condition over the 120-min period for which the trajectories of the ALDs were simulated. It is an ongoing point of research to determine if the effects of adding the time-dependent component to the supercell simulation would result in a large enough change of the trajectories to warrant the added computational cost of running such large datasets.

The ALD system has been flown on the Mistral in a number of promising test flights. As of August 2018, one successful release of an ALD balloon in flight had been achieved. With further testing, it is believed that dependable ALD release will be possible by the end of the year for deployments in the spring of 2019. 

## Figures and Tables

**Table 1:** MS8607 [12] PTH sensor specifications.

	Max. Operating Range	Accuracy	Resolution
**Pressure**	10–2000 mbar	±2 mbar	0.016 mbar
**Temperature**	−40–80 ${}^{\circ }$C	±1 ${}^{\circ }$C	0.01 ${}^{\circ }$C
**Relative Humidity**	0–100%	±3%	0.01%

**Table 2:** GPS Module ublox CAM-M8Q [14] specifications.

Horizontal Position Accuracy	Maximum Navigation Update Rate	Sensitivity
2.5 m	10 Hz	−166 dBm

**Table 3:** Air-launched drifter (ALD) mass distribution.

Component	Mass
Microsonde	17.0 g
Mylar Balloon	48.0 g
Connectors	0.6 g
**Total:**	**65.6 g**
